# Identification of potential impacts of climate change and anthropogenic activities on streamflow alterations in the Tarim River Basin, China

**DOI:** 10.1038/s41598-017-09215-z

**Published:** 2017-08-15

**Authors:** Lianqing Xue, Fan Yang, Changbing Yang, Xinfang Chen, Luochen Zhang, Yixia Chi, Guang Yang

**Affiliations:** 1 Hydrology and Water Resources College, Hohai University, Nanjing, 210098 P.R. China; 2 Jackson School of Geosciences, University of Texas at Austin, Austin, 78712 USA; 3 Shihezi University, Shihezi, 832003 P.R. China; 4 Tarim River Basin Administration, Korla, 841000 P.R. China; 5 Hohai University Wentian College, Maanshan, 243000 P.R. China

## Abstract

Understanding contributions of climate change and human activities to changes in streamflow is important for sustainable management of water resources in an arid area. This study presents quantitative analysis of climatic and anthropogenic factors to streamflow alteration in the Tarim River Basin (TRB) using the double mass curve method (DMC) and the Budyko methods. The time series (1960~2015) are divided into three periods: the prior impacted period (1960~1972) and the two post impacted periods, 1973~1986 and 1987~2015 with trend analysis. Our results suggest that human activities played a dominant role in deduction in the streamflow in TRB with contribution of 144.6% to 120.68% during the post impacted period I and 228.68% to 140.38% during the post impacted period II. Climatic variables accounted for 20.68%~44.6% of the decrease during the post impacted period I and 40.38% ~128.68% during the post impacted period II. Sensitivity analysis indicates that the streamflow alteration was most sensitive to changes in landscape parameters. The aridity index and all the elasticities showed an obvious increasing trend from the upstream to the downstream in the TRB. Our study suggests that it is important to take effective measures for sustainable development of eco-hydrological and socio-economic systems in the TRB.

## Introduction

Change in climate conditions and anthropogenic activities contribute to significant alterations in the eco-hydrological patterns in many basins world widely^1–8^. Such alterations further lead to changes of hydrological processes, causing severer water issues, especially in arid regions and also irreversible environmental consequences to riverine ecological systems including dwindling in lake areas, degradation in water ecosystems and loss/fragmentation of natural habitats^9–13^. Therefore, it is particularly important to assess responses of hydrological systems to climate change and human activities to improve our understanding of the hydrological processes and to develop scientific-based strategies of sustainable water resources management and protection.

Many efforts have been made in the last decades to identify the impacts of climate change and human activities on streamflow alterations^9, 14–20^. Ahn and Merwade (2014) analyzed climate and human impacts on streamflow conditions using historical streamflow records in Indiana, New York, Arizona and Georgia in USA, in conjunction with trend analysis and hydrologic modeling. The researchers reported that the human impact is higher on streamflow at most gauging stations in all four states compared to climate impact^14^. Ma *et al*. (2010) reported a case study to distinguish impacts of climate variability and human activity on stream flow decrease in Miyun Reservior catchment over a period of 50 years using a distributed hydrological model and a climate elasticity model and reported that climate impact was accountable for about 55% while human activity (mainly manmade land use and vegetation changes) accounted for 18% of the decrease in reservoir inflow^21^.

The impacts of human activity and climate change on streamflow in an arid area seems apparently significant^22^. The objective of this study is to identify factors that caused streamflow alterations on the Tarim River, which is the longest continental river in the world, located in the arid area of north-western China. The Tarim River Basin (TRB) has been experienced water scarcity, resulting in conflicts among water consumers at upstream and downstream regions, and degradation of its natural ecosystems^23, 24^. The population growth, irrational exploitation and utilization of water resources, such as irrigation and dam construction, have significantly changed functions of river ecosystem^10–12^.

While various methods have been presented in literature to distinguish impacts of climate change on streamflow alterations from impacts of human activities, one approach widely used is to combine trend analysis with empirical statistical approaches, such as the Mann–Kendall test because of the simplicity^17, 25^. Empirical statistical approaches usually establish the relationship between streamflow and climatic factors based on the historical hydro-meteorological data series^8, 15^. The empirical statistical approach may also be combined with the Double Mass Curve and the Budyko analysis, providing a more efficient way in analyzing streamflow alterations from hydro-meteorological data series^8, 14, 26–30^. The Budyko analysis was proposed on the assumption that the ratio of evaporation to precipitation is controlled by the ratio of potential evapotranspiration (*ET*_*P*_) to precipitation. It has shown that the combined framework could provide assessment as reliable as the complex hydrological models^31, 32^. Some physical based hydrological models, such as physically-based distributed MIKE SHE model^33^, generalized additive model^34^, Variable Infiltration Capacity^35^, Soil and Water Assessment Tool^36^ are also used. However, these hydrological models generally require various datasets which are typically lack at the TRB. To improve our understanding of the impacts of climatic and anthropogenic factors on streamflow alteration in the TRB, this study aims: (1) to detect statistically variable trends and the change point in hydro-meteorological series; (2) to explore contributions and sensitivity of climate change and human activities to streamflow variability with the double mass curve method and the elasticity method based on the Budyko equations; and (3) to analyze elasticity of streamflow to precipitation, potential evapotranspiration and landscape.

## Results

### Variation of hydro-meteorological variables

**Figure 1:**
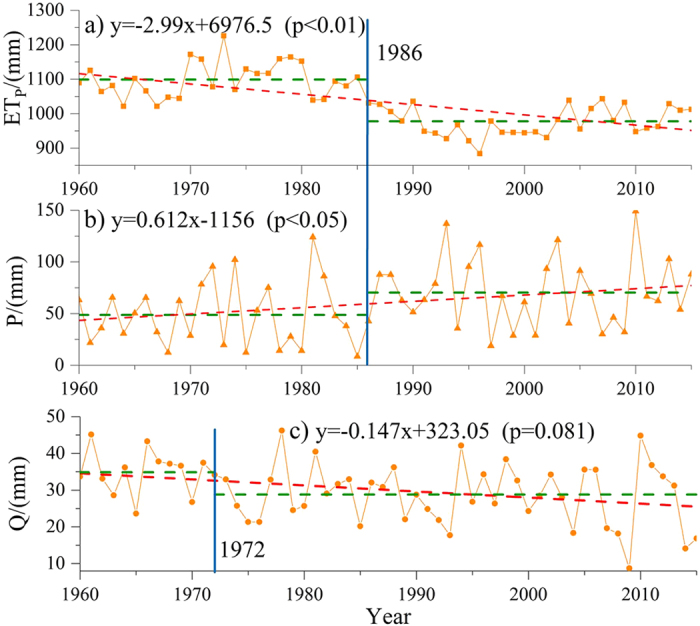
The trend of annual potential evapotranspiration, precipitation, streamflow at the Alar station in the Tarim River Basin during 1960~2015 (Note: *P*, *ET*_*P*_, *Q* indicate the precipitation, potential evapotranspiration and streamflow, respectively. Green dotted lines represent the average values during the prior impacted period and post impacted period, Red dotted lines represent annual variation tendency of the sequence. *p* value is the significance level of Pettitt-test).

The trends in precipitation, evapotranspiration and streamflow at the Alar station during 1960~2015 are shown in Fig. 1. Precipitation shows a slight upward trend with a slope of 0.61 mm/year over a period of 1960 to 2015 (R^2^ = 0.10). Potential evapotranspiration shows a significant decreasing trend with a slope of −2.99 mm/year (R^2^ = 0.41). Similarly, streamflow shows a slight decreasing trend with a slope of −0.15 mm/year (R^2^ = 0.08).

**Figure 2:**
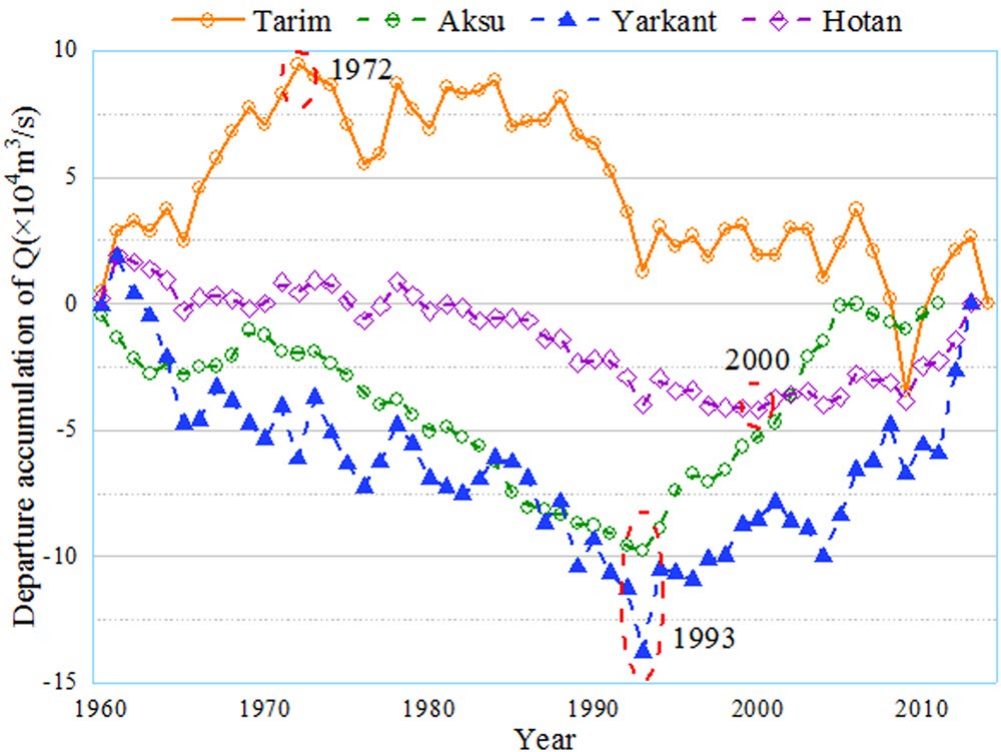
Comparisons of change point of streamflow between the Tarim headstreams and the mainstream during 1960~2015 (Note: lines represent the departure accumulation of streamflow with cumulative average deviation method. The streamflow in headstreams are observed at the Shaliguil in Aksu, the Kaqun in Yarkant and the Wuluwati in Hotan River, respectively. The streamflow in mainstream is observed at the Alar in the Tarim River).

The Mann-Kendall test and Pettitt test were used to examine the trends in the annual runoff, precipitation and potential evapotranspiration series of 1960~2015 and determine change points of time series of precipitation, potential evapotranspiration and streamflow at the Alar station (Fig. 1). The change points occurred in 1986 (p < 0.05) firstly for precipitation and potential evapotranspiration at most of Meteorological stations within the basin (Fig. 1a,b, Table 1). Unlike precipitation and potential evapotranspiration, streamflow shows the change point in 1972 at a significance level of 0.081 (Fig. 1c). Meanwhile, the cumulative departure curve of streamflow illustrated the turning point for streamflow is 1972 in the Tarim river during the period of 1960~2015, while the turning point for streamflow is 1993, 1993 and 2000 in headstreams within the basin (Fig. 2). The inconsistency between headstreams and mainstreams well reflects the fact that human activities have severely disturbed water distribution over time and space. The streamflow alterations were divided into three periods: the prior impacted period (1960~1972), the post impacted period I (1973~1986) and the post impacted period II (1987~2015). Table 2 lists estimation of change points for precipitation, annual potential evapotranspiration, and streamflow with the four methods. It can be seen that the four methods provide similar estimation for the change points. Streamflow alterations during the post impacted period were likely caused by human activities, such as irrigation and dam construction^11, 12^. During the post impacted period II, a warmer and wetter climate together with over exploitation of water resources in the TRB likely caused streamflow alterations^23^.

**Table 1:** Summary statistics for the annual precipitation and potential evapotranspiration analysis at 13 stations in the Tarim River Basin during 1960~2015.

Meteorological station	Annual precipitation			Annual potential evapotranspiration		
	Abrupt change	Trend	Significance	Abrupt change	Trend	Significance
Aheqi	1990	↑	*	1986	↓	**
Aksu	1991	↑	*	1987	↓	**
Bachu	1986	↑	*		/	
Baicheng	1986	↑	**	1986	↓	**
Kuche	1987	↑	*	1986	↓	**
Kuerle	1986	↑	*	1986	↓	**
Minfeng	1986	↑	*	1987	↓	**
Pishan	1986	↑	*	1986	↓	**
Ruoqiang		/		1986	↓	**
Shache	1986	↑	*		/	
Tashikuerg	1987	↑	*	1986	↓	*
Tieganlik		/		1986	↓	**
Yutian	1986	↑	*		/	

Notes: In the Pettitt test, **indicates significant level of 0.1%, *indicates significant level exceeds 5%, ↑(↓) indicates an increasing(decreasing) trend, and/indicates unremarkable change trend.

**Table 2:** The change point of meteorological and hydrological factors in the Tarim River Basin during 1960~2015.

Hydro-meteorological data	Mann-Kendall test	Cumulative average deviation	Pettit test	Sequential cluster method
Annual precipitation	1987	1987	1986	1986
Annual potential evapotranspiration	1986	1986	1986	1990
Annual streamflow	1973	1972	1972	1972

### Relative contribution of climate change and human activities on streamflow alteration

**Figure 3:**
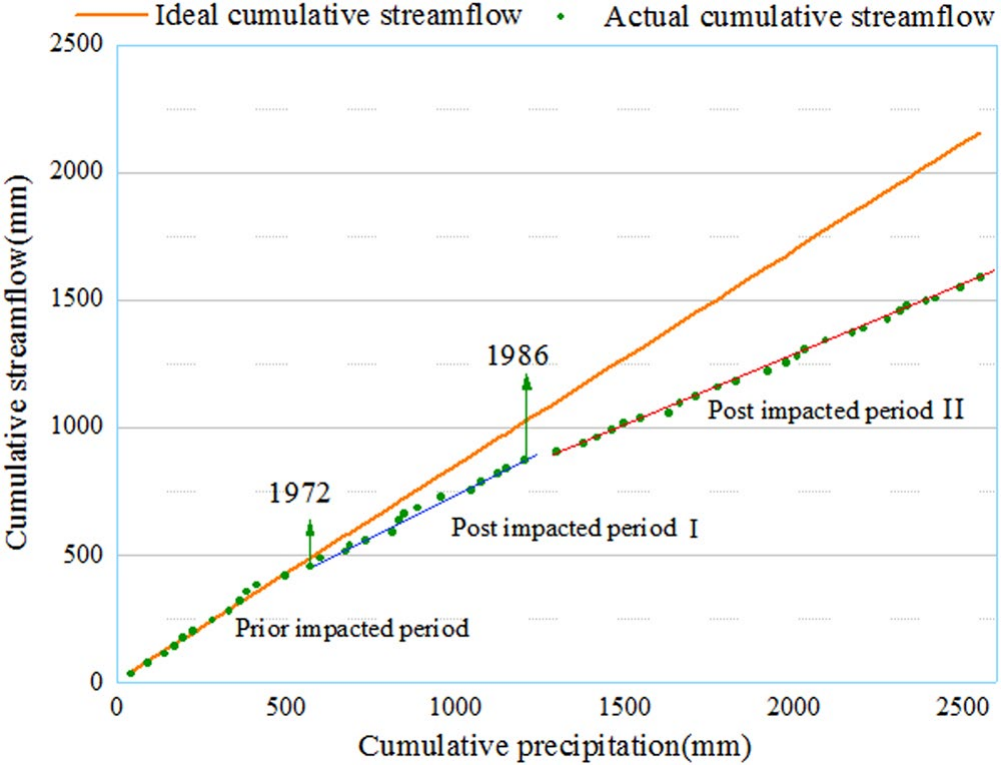
The plot of cumulative precipitation versus cumulative streamflow with the Double mass curve analysis of the Tarim River during 1960 to 2015.

DMC and three Budyko equations were used to quantify the roles of climatic and anthropogenic factors on streamflow alteration during the period of 1973–2015 in the TRB. Figure 3 shows the relationship between accumulated precipitation and streamflow during the prior impacted period and the two impacted periods. Cumulative streamflow apparently deviates from the cumulative rainfall since 1972 (Fig. 3). The relationship between accumulative precipitation and accumulative streamflow during the prior impacted period could be fitted linearly by the following equation,

1 $${\sum }^{}Q=0.843{\sum }^{}P+3.6423$$

The correlation coefficient is approximately 0.9864.

**Figure 4:**
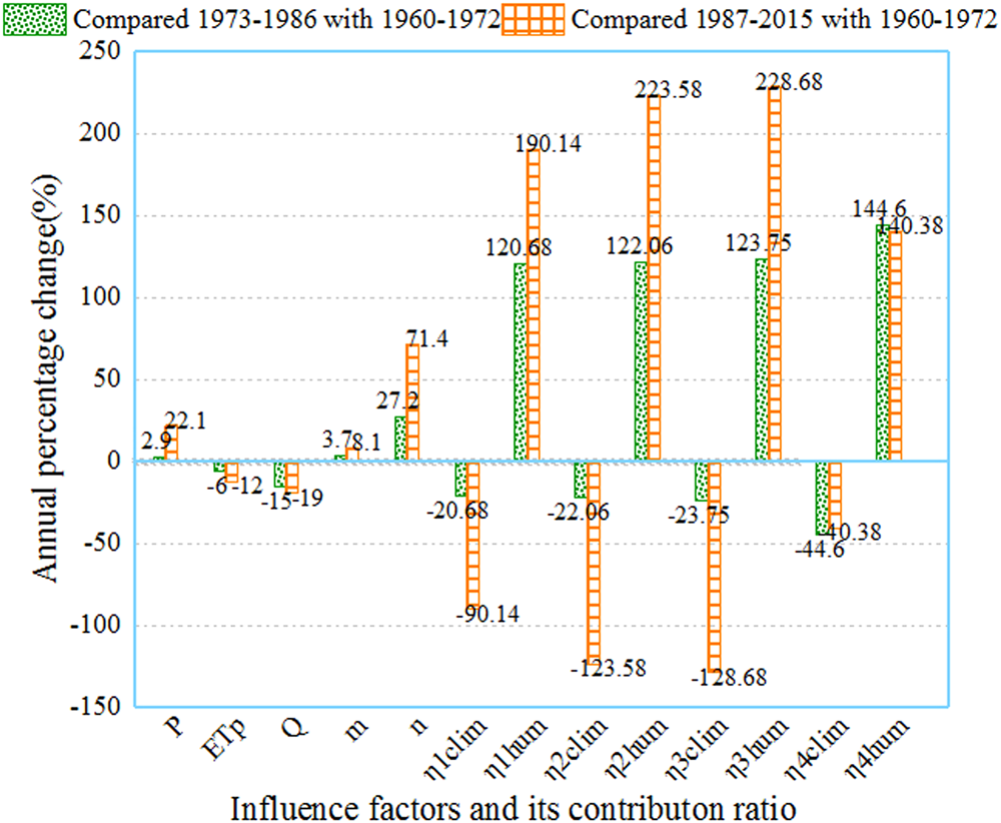
Barplot of annual runoff change in different periods (Note: $\eta {1}_{clim}$, $\eta {2}_{clim}$, $\eta {3}_{clim}$, $\eta {4}_{clim}$ are the contribution rates of climate change in Budyko, Fu. Choudhury equations and DMC, respectively. $\eta {1}_{hum}$, $\eta {2}_{hum}$, $\eta {3}_{hum}$, $\eta {4}_{hum}$ are the contribution rates of human activities in Budyko, Fu. Choudhury equations and DMC, respectively).

**Figure 5:**
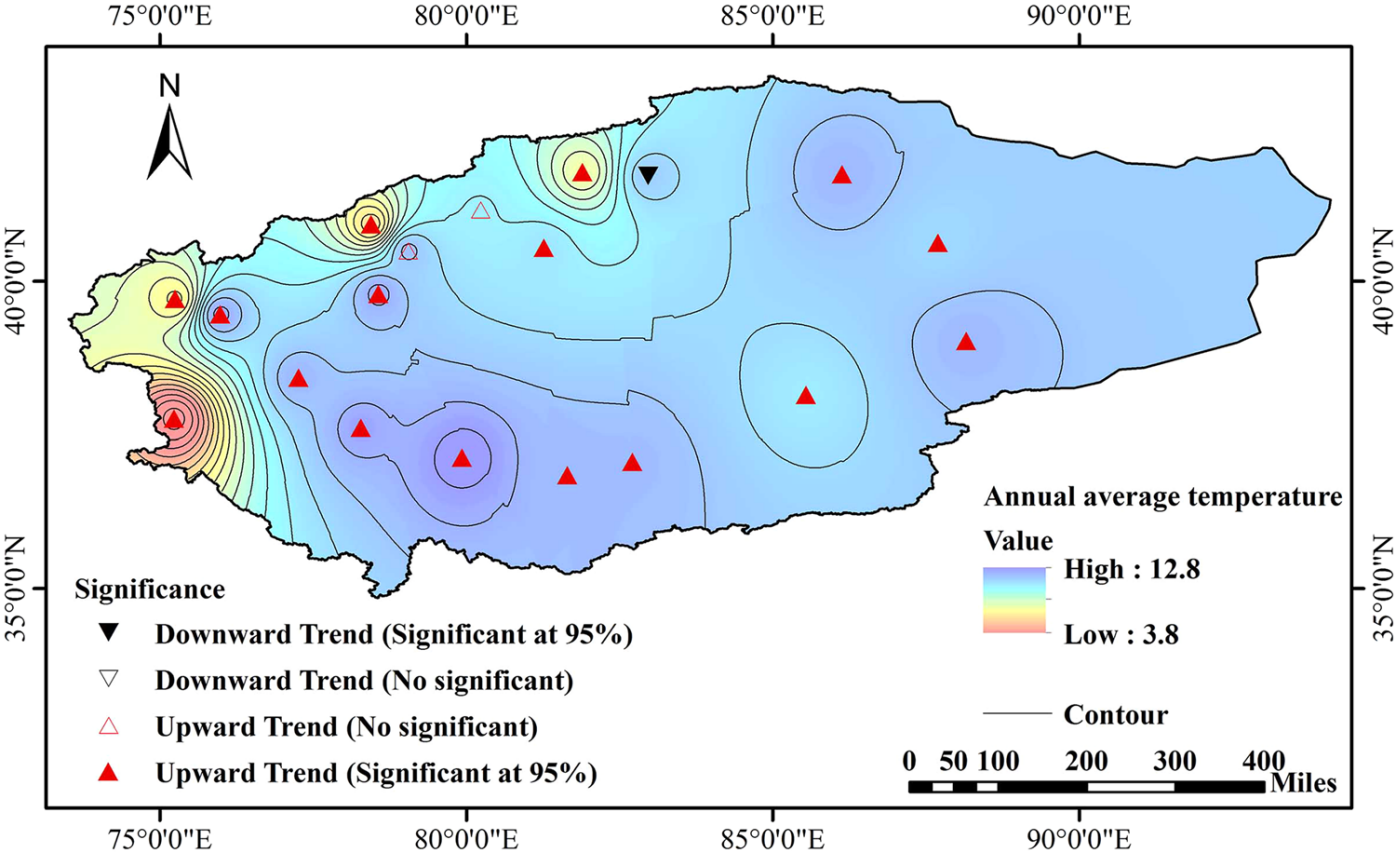
Spatial distribution of annual average temperature and trends of annual temperature measured by the Mann-Kendall test in the TRB (Note: the map was generated with data available from China Meteorological Data Sharing Service System (http://data.cma.cn) and the Chinese Geospatial Data Cloud using ESRI’s ArcGIS (version 10.1; http://www.gscloud.cn/).

Quantitative estimation for contribution of climatic change and human activity to streamflow alterations is given in Table 3. Note that a positive percentage value represents a beneficial impact that increase streamflow while a negative percentage value is for negative impact which decreases streamflow. The results of three Budyko equations showed that climate change accounted for −20.68~−23.75% and human activity for 123.75~120.68% during the post-impacted period I. During the post impacted period II, contribution of climate change to streamflow alteration increased to −90.14~−128.68%, while the negative impact of human activity on streamflow alterations increased to 228.68~190.14%. Similarly, The DMC indicated that contribution of human activities accounted for 144.60% of the total deduction in the streamflow during the post impacted period I and for 140.38% during the post impacted period II (Fig. 4). Apparently the Budyko and DMC methods leads to a consistent conclusion that human activity played a major role in streamflow deduction during the post impacted periods. In addition, climate change contributed a positive impact on streamflow in the TRB likely because increase in temperature caused more snow melting which were accumulated at the mountainous areas (Fig. 5).

**Table 3:** Contributions of climate variability and human activities on streamflow alterations.

Period	P(mm)	ET_p_(mm)	Q(mm)	m	n	$\eta {1}_{clim}$	$\eta {1}_{hum}$	$\eta {2}_{clim}$	$\eta {2}_{hum}$	$\eta {3}_{clim}$	$\eta {3}_{hum}$	$\eta {4}_{clim}$	$\eta {4}_{hum}$
1960–1972	38.7	2176.18	35.53	1.04	0.22								
1973–1985	47.71	2084.82	30.22	1.08	0.28	−20.68%	120.68%	−22.06%	122.06%	−23.75%	123.75%	−44.60%	144.60%
1986–2011	53.87	1924.68	28.76	1.13	0.37	−90.14%	190.14%	−123.58%	223.58%	−128.68%	228.68%	−40.38%	140.38%

Note: $\eta {1}_{clim}$, $\eta {2}_{clim}$, $\eta {3}_{clim}$, $\eta {4}_{clim}$ are the contribution rates of climate change in Budyko, Fu., Choudhury equations and DMC, respectively. $\eta {1}_{hum}$, $\eta {2}_{hum}$, $\eta {3}_{hum}$, $\eta {4}_{hum}$ are the contribution rates of human activities in Budyko, Fu., Choudhury equations and DMC, respectively.

### Sensitivity of streamflow alterations to *P, ET*_*P*_*, n*

Table 4 lists the elasticity of streamflow alterations on climate variables and human activities. During the post impacted period II, the elasticities of streamflow to *P* and *ET*_*P*_ are 1.19 and −0.19 on average, respectively, indicating that a 1% increase in *P* (or *ET*_*P*_) would increase streamflow by 1.19% (or decrease streamflow by 0.19%). The elasticity of streamflow to parameter *n* is −1.93 on average, implying that a 1% increase in *n* would decrease streamflow by 1.93%. Our results show that streamflow alteration is the most sensitive to catchment landscape parameter (*n*), followed by changes in precipitation and potential evapotranspiration in the TRB. Note that streamflow is positively correlated with precipitation but negatively with potential evapotranspiration and catchment landscape parameter.

**Table 4:** The elasticity of streamflow alterations on climate variability and human activities.

Period	Budyko		Fu *et al*.			Choudhury		
	${{\boldsymbol{\varepsilon }}}_{{\boldsymbol{P}}}$	${{\boldsymbol{\varepsilon }}}_{{\boldsymbol{E}}{{\boldsymbol{T}}}_{{\boldsymbol{P}}}}$	${{\boldsymbol{\varepsilon }}}_{{\boldsymbol{P}}}$	${{\boldsymbol{\varepsilon }}}_{{\boldsymbol{E}}{{\boldsymbol{T}}}_{{\boldsymbol{P}}}}$	${{\boldsymbol{\varepsilon }}}_{{\boldsymbol{m}}}$	${{\boldsymbol{\varepsilon }}}_{{\boldsymbol{P}}}$	${{\boldsymbol{\varepsilon }}}_{{\boldsymbol{E}}{{\boldsymbol{T}}}_{{\boldsymbol{P}}}}$	${{\boldsymbol{\varepsilon }}}_{{{\boldsymbol{n}}}_{{\bf{0}}}}$
1960–1972	2.999	−1.999	1.044	−0.044	−0.501	1.061	−0.061	−0.791
1973–1986	2.998	−1.998	1.082	−0.082	−1.253	1.127	−0.127	−0.992
1987–2015	2.999	−1.999	1.128	−0.128	−1.975	1.261	−0.261	−1.884

The elasticity coefficients estimated with the three Budyko equations show some differences (Table 4). Precipitation change-induced streamflow increased from 1.044 for the Fu equation to 2.999 for the Budyko equation. The catchment landscape elasticity ranged from −1.975 for the Fu equation to −0.501 (Fu). It indicates that the elasticities in Budyko changes slightly from prior impacted period to post impacted period, while the elasticities between Fu and Choudhury equation are consistent with variation.

**Figure 6:**
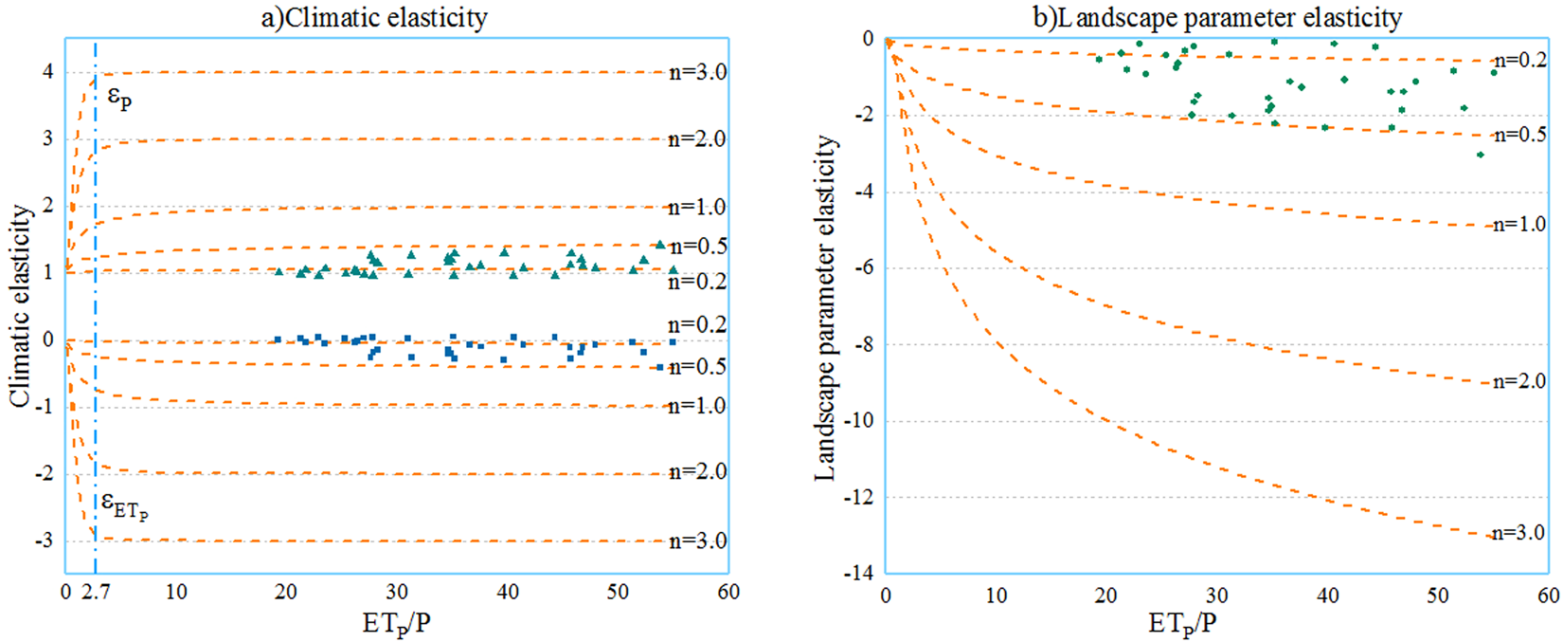
Relationship between the climate elasticity/landscape elasticity of streamflow and the aridity index (Note: the lines represents the elasticity of streamflow, the green triangles represent ${{\rm{\varepsilon }}}_{P}$, the blue dots represent ${{\rm{\varepsilon }}}_{E{T}_{P}}$ and the green squares represent ${{\rm{\varepsilon }}}_{{n}_{0}}$ in the TRB).

Figure 6 shows the relationship between the climate and landscape elasticity with the aridity index and the landscape parameter using the Choudhury equation. The precipitation elasticity in our study area ranges from 1.00 to 1.53 and the potential evapotranspiration elasticity ranges from −0.53 to 0.00, while the landscape elasticity changes from −3.03 to −0.09. Results suggest that climate and landscape elasticity showed an increasing trend with the increase of aridity index. However, when the aridity index increased to 2.7, the climate elasticity shows a weakly increasing trend (Fig. 6a), the landscape elasticity remain increasing significantly (Fig. 6b). It suggests that all elasticities in arid regions are more sensitive to runoff changes than humid regions.

**Figure 7:**
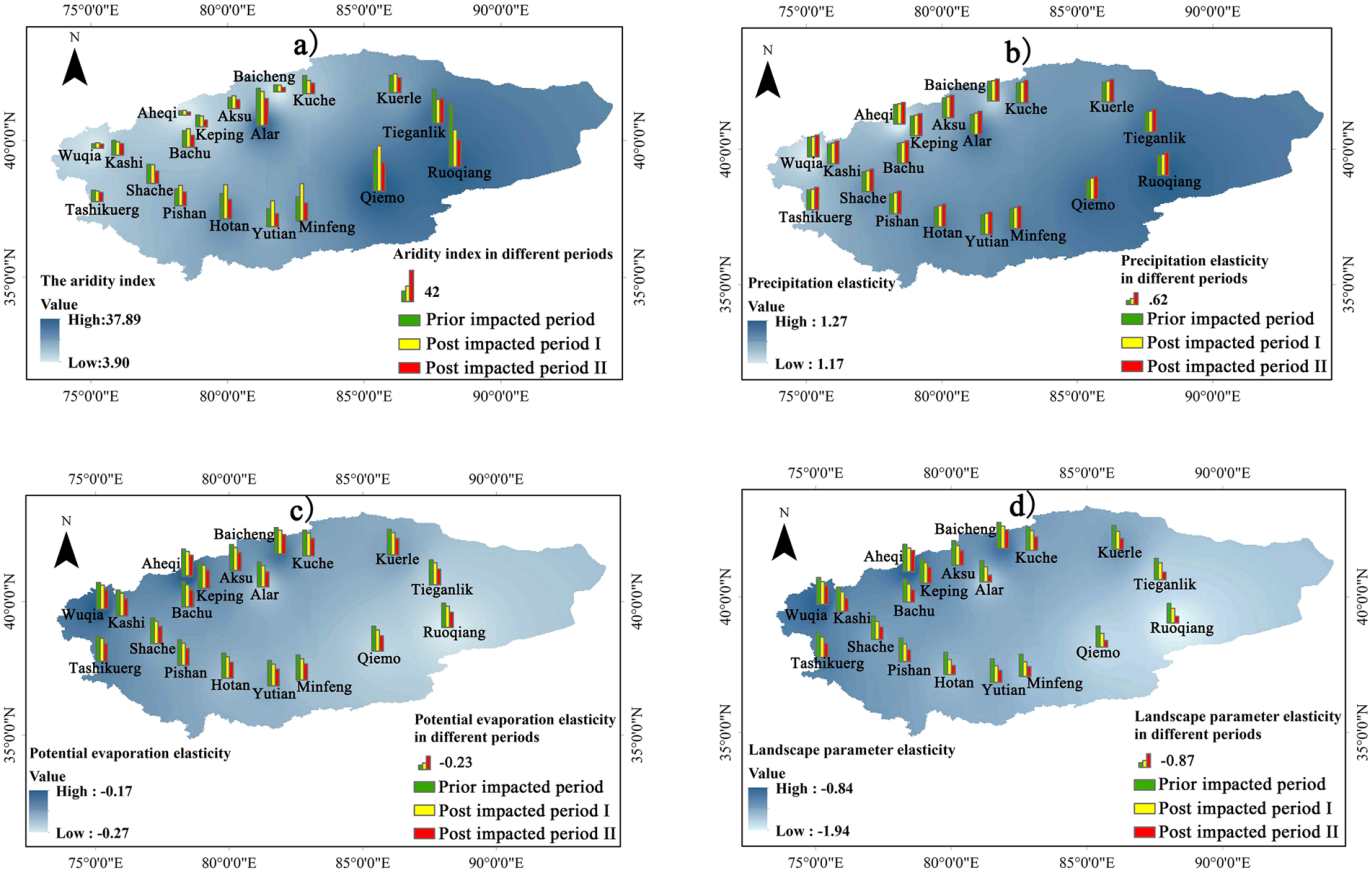
Plots of spatial distributions of the aridity index and the elasticities of streamflow in the Tarim River Basin in different period (Note: The map was generated with data available from the Chinese Geospatial Data Cloud using ESRI’s ArcGIS (version 10.1; http://www.gscloud.cn/). (**a**,**b**,**c**,**d**) Represent the aridity index, precipitation elasticity, potential evapotranspiration elasticity, and landscape parameter elasticity, respectively. The blue gradient mask represents the spatial distribution of the aridity index (or precipitation elasticity/potential evapotranspiration elasticity/landscape parameter elasticity) in post impacted period II).

The temporal variations and spatial distributions of aridity index, precipitation elasticity, potential evapotranspiration elasticity and landscape parameter elasticity are respectively analyzed over nineteen stations as well as for the TRB. As shown in Fig. 7, a significant increase from 3.90 to 37.89 occurred in aridity index from the upstream to the downstream in the TRB. Similarly, the elasticity of precipitation, potential evapotranspiration and landscape parameter showed an obvious increasing trend. In particular, the landscape parameter elasticity ranged from −0.84 to −1.94 from the upstream to the downstream in the TRB. On the other hand, all the elasticities increased slightly while the aridity index decreased significantly.

## Discussion

Our results conclusively reveal that human activity was the dominant factor to streamflow alteration in the TRB during the post impacted period I and both climate change and human contributed to streamflow alteration during the post impacted period II. Moreover, the elasticity of precipitation, potential evapotranspiration and landscape parameter showed a significant increasing trend from prior impacted period to post impacted period II, and streamflow is more sensitive to landscape parameter. That is to say, along with the intensification of the human activity, the impact of it became more dominant in the alteration of streamflow.

As for human activity, cultivated area expansion and agricultural irrigation, dam construction are the respective factors in the TRB. Oasis agriculture is the principal economy in the basin and agricultural water consumption accounts for a large proportion of the total water consumption. Therefore, changes in cultivated land area can prove the extent of human impacts on water resources. Land use and cover change can describe the impact of oasis extension and agricultural irrigation on streamflow clearly. The farmland, forest land and residential land increased heavily while the grass land decreased. It implies that some parts of grass land transformed into mudflat and reed land farmland, forest land and residential land (Fig. S1 in the Supporting information). Compared with 1980, the area of farmland, forest land and residential land increased by 51.84%, 19.53% and 40.14% in 2015, respectively (Table 5). In contrast, the area of grass land decreased 4.81%. The dramatic change indicated that the demand of agricultural irrigation from oasis extension was obviously deleterious to water high-efficiency and reasonable utilization of water resources. In fact, oasis water consumption has been persistently increasing from 1960 to 2011 in the TRB (Fig. S2c in the Supporting information). Moreover, the government statistics show that the area of actual irrigation exceeds area of government-allowed irrigation by as much as 4 × 10^5^ hm^2^. It indicates that more and more ecological water is consumed for the excessive expansion of irrigated area and agricultural water consumption is still climbing at an alarming rate despite the gradually improved water use efficiency and reformed irrigation management. Meanwhile, reservoir construction has become great challenges for the sustainable development of water resources in the TRB. Currently, the 76 plain reservoirs with a total storage capacity of 2.8 × 10^9^ m^3^ affect natural runoff distribution and change the flood peak discharge. For example, to satisfy the ecological and agricultural water use in the mainstream in the TRB, water managers regulated the streamflow in the low-flow period by implementing water conservation projects. Plain reservoirs are shallow with a relatively large water surface, thus leading a large amount of evaporation in the arid area. In addition, water seepage losses is huge for unlined irrigation canals and soil-based reservoirs, resulting in waterlogging and salinization due to the raise of the groundwater table. Figure S2b shows that water consumption taken from the three great plain reservoirs during 1981 to 2006 were significantly increased. However, the ratio of water consumption of three plain reservoirs in total water consumption showed a decreasing trend (Fig. S2a in the Supporting information), it indicates that other ways of water consumption played a more dominant role in the alteration of streamflow. Therefore, it is unequivocally clear that a total amount of decreased water resources within TRB in the past half century have been mainly utilized for implementing water conservation projects and expanding irrigated areas. The eco-environment with TRB has been deteriorating in recent years, especially in the downstream of the basin. Signs of deterioration in the eco-environment warm us that human activities including irrationally water resources utilization have exceeded the environmental carrying capacity, and even destroyed the integrity of the natural ecosystems.

**Table 5:** Change of land use and vegetation cover in the Tarim River Basin in 1980, 1990, 2000, 2008, and 2015.

Year	Farmland		Forest land		Grass land		Water		Residential land		Unulitized land	
	Area (km^2^)	Rate (%)	Area (km^2^)	Rate (%)	Area (km^2^)	Rate (%)	Area (km^2^)	Rate (%)	Area (km^2^)	Rate (%)	Area (km^2^)	Rate (%)
1980	24502		10672		260551		36694		1607		647064	
1990	24503	*0.00*	10674	*0.02*	260526	−0.01	36544	−0.41	1608	*0.06*	647075	0.00
2000	26930	*9.91*	13806	*29.37*	253732	−2.62	37292	1.63	1648	*2.55*	648079	0.16
2008	31684	*29.31*	13163	*23.34*	251367	−3.52	36775	0.22	1710	*6.41*	646588	−0.07
2015	37203	*51.84*	12756	*19.53*	248021	−4.81	36767	0.20	2252	*40.14*	644288	−0.43

Climate variability is an important factor to streamflow alteration. The uneven distribution of rainfall, evaporation and temperature variation may affect the temporal and spatial characteristics of water resources. In fact, the present study indicated that the change point of climatic variables in the TRB is 1986. Temperature and precipitation increased significantly and potential evapotranspiration showed a decreasing trend. Further investigation can be focused on climate change effects on snow accumulation (thickness) and snowmelt mechanism in the TRB.

In view of the slow and partly uncontrollable influence of climate-induced changes, effective measures should be taken to restrain impacts of human activities on streamflow alterations in order to restore ecological integrity in the TRB. Three suggestions could be followed. The first suggestion is to restrict human activity to a reasonable range. The policy of reasonable allocation of water resources in the TRB can be implemented to alleviate conflicts between the upstream and downstream regions. Reservoir operation should ensure water release and storage capacity of aquatic ecosystems and maintain the ecological integrity. The second suggestion is to design an integrated modeling framework by combining groundwater planning and management together, providing guidance to recover the hydrological pattern in the TRB. The third suggestion is to establish demonstration zones at local levels to obtain experiences for water resources management and utilization and then expanded to the whole basin.

## Materials and Methods

### Study area

The TRB, located in an extremely arid area of Northwest China, and spans 34°55′~43°08′N and 73°10′~94°05′E, with a catchment area of 1.02 × 10^6^ km^2^, is the longest inland river in the world^37^. The Tarim River is mainly fed by glacier and snow-melt water from the Tianshan Mountain and the Kunlunshan Mountain and precipitation. Flow is dissipated in the desert area of the oasis and disappears in the desert area.

**Figure 8:**
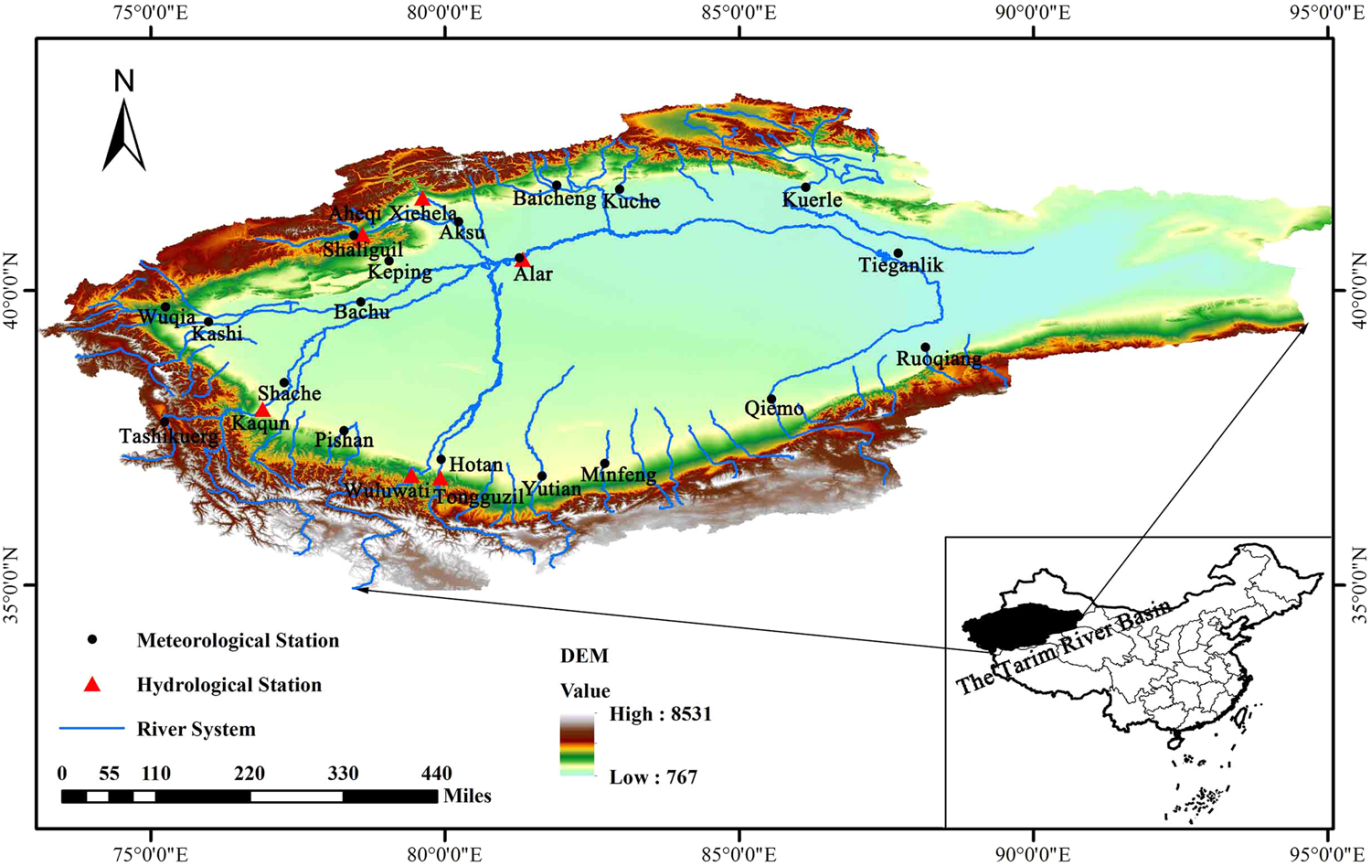
Sketch map of the Tarim River basin and location of the hydro-meteorological stations (Note: the map was generated with data available from the Chinese Geospatial Data Cloud using ESRI’s ArcGIS (version 10.1; http://www.gscloud.cn/).

There are three main headwater streams that supply water to the main stream, including the Aksu River, the Hotan River and the Yarkant River. These rivers feed the TRB at the Alar Gauge Station (Fig. 8) with the portions of water discharge 73.2%, 23.2%, and 3.6% measured at the Alar Gauge Station. In recent years, dam constructions and agricultural irrigation have had substantial impact on sustainable development of water resources in such arid regions (Fig. S2 in the Supporting information). The socioeconomic development has led to a rapid change in land use in the TRB over recent decades (Fig. S1 in the Supporting information).

### Data

Figure 8 shows locations of the six hydrological stations and nineteen meteorological observatory stations in the TRB. Annual streamflow data were collected from the six hydrological stations (Xiehela and Shaliguil in Aksu River; Kaqun in the Yarkant River; Tongguzlok and Wuluwati in the Hotan River) over a period of 1960 to 2015. The meteorological data (daily precipitation, air temperature, sunshine hours, relative humidity and wind speed) over the period of 1960 to 2015 were obtained from the China Meteorological Data Sharing Service System (http://data.cma.cn). The potential evapotranspiration was calculated using the Penman model^38^. Land use and vegetation data about 1980, 1990, 2000, 2008 and 2015 were collected from Resources and environment science data center in Chinese academy of sciences (http://www.resdc.cn). Oasis water consumption data from 1960 to 2011 and water consumption data taken from the three great plain reservoirs during 1981 to 2006 were obtained from the Tarim River Basin Administration.

## Methods

### Trend test

Four methods, the Mann-Kendall test, Pettitt’s test^39^, the cumulative departure curve and sequential cluster method^40^ are used for determining the abrupt change point to reducing errors or leakage test from a single method^41, 42^. The Mann–Kendall test, a rank-based nonparametric method for detecting time series trends without requiring normality or linearity, has been widely applied to assess trends in hydro-meteorological time series, such as streamflow, precipitation and temperature^43, 44^. The readers who are interested in the details of the four methods may refer to the literature provided here^39–42^.

### Double-mass curve method

The double-mass curve (DMC) is one of the most useful techniques in testing the consistency and trends in long term hydro-meteorological data^15^. The method uses linear regression analysis of time series. The relationship between cumulative runoff and cumulative precipitation in the prior impacted period can be described as:

2 $$\sum _{i=1}^{t}{Q}_{i}^{baseline}=a\sum _{i=1}^{t}{P}_{i}^{baseline}+b\,t=1,2,3,\cdots ,n$$

where *a* denotes the rate of change in cumulative runoff with changes in accumulated precipitation, *b* is the intercept, and *n* is the length of the annual time series. If there are no errors or changes in disturbance of human activities, all points will fall (approximately) on this straight-line, and *a* is a constant. Once there is an interference of human activities and climate change, the regression equation is used to simulate streamflow in the post impacted period:

3 $$\sum _{i=1}^{t}{Q}_{i}^{variation}=a\sum _{i=1}^{t}{P}_{i}^{variation}+b\,t=1,2,3,\cdots ,n$$

In the current study, cumulative rainfall is described as X axis and cumulative runoff is shown in Y axis. Contribution to changes in streamflow due to human activities can be calculated:

4 $${\rm{\Delta }}{\bar{Q}}_{hum}={\bar{Q}}_{variation}-{\bar{Q}}_{construct}$$

and contribution to changes in streamflow caused by climate change is given by

5 $${\rm{\Delta }}{\bar{Q}}_{clim}={\rm{\Delta }}{\bar{Q}}_{tot}-{\rm{\Delta }}{\bar{Q}}_{hum}$$

where ${\bar{Q}}_{construct}$ and ${\bar{Q}}_{variation}$ represent the average of the reconstructed runoff data and the observed runoff data during the post impacted period. ${\rm{\Delta }}{\bar{Q}}_{tot}$ is the total changes in streamflow.

### Budyko-based Equations

In an ideal basin, the water balance for a closed catchment can be written as:

6 $$P=E+Q+{\rm{\Delta }}S$$

where *P*, *E* and *Q* are precipitation, actual evapotranspiration and streamflow, Δ*S* is the change in catchment water storage. Over a long period (e.g. 5 and 10 years), ΔS can be assumed to be zero. Under the influence of climate-induced changes and anthropogenic perturbations, streamflow variation can be assumed as:

7 $${\rm{\Delta }}{Q}_{tot}=|{\bar{Q}}_{variation}-{\bar{Q}}_{baseline}|$$

where ${\rm{\Delta }}{Q}_{tot}$ is total change of streamflow, ${\bar{Q}}_{variation}$ and ${\bar{Q}}_{baseline}$ denote streamflow in post impacted and prior impacted period, respectively ${\rm{\Delta }}{Q}_{tot}$ can be given by:

8 $${\rm{\Delta }}{Q}_{tot}=|{\rm{\Delta }}{\bar{Q}}_{clim}+{\rm{\Delta }}{\bar{Q}}_{hum}|$$

Thus the relative contribution to runoff change by each factor is calculated as:

9 $${\eta }_{c}=\frac{{\rm{\Delta }}{\bar{Q}}_{clim}}{{\rm{\Delta }}{Q}_{tot}}\times 100 \% \,{\eta }_{h}=\frac{{\rm{\Delta }}{\bar{Q}}_{hum}}{{\rm{\Delta }}{Q}_{tot}}\times 100 \% $$

where *η*_*c*_ and *η*_*h*_ are contribution of change in climatic factors and human activities, respectively.

The long-term mean annual *E* is mainly determined by *P* and $E{T}_{p}^{45}$. Following the assumption by MI Budyko^46^, the actual evaporation can be estimated:

10 $$E/P=F(\phi )$$

where $\phi =E{T}_{p}/P$, *φ* is aridity index and division basis between climatic zone and natural vegetation zone. Taking into account of the relationship between mean annual *P*, *ET*_*p*_ and *E*, *Q* can be written as a function of climatic variable and catchment characteristics:

11 $$Q=f(P,E,n)$$

The parameter *n* represents the catchment characteristics connected with soil type, topography and vegetation. In the study, we assumed that changes in *n* over periods are primarily caused by anthropogenic activities and can reflect the impacts of anthropogenic activities on *Q* change. Then variation in runoff caused by changing climate and catchment characteristics can be approximated as:

12 $$\frac{dQ}{Q}={{\rm{\varepsilon }}}_{P}\frac{dP}{P}+{{\rm{\varepsilon }}}_{E{T}_{p}}\frac{dE{T}_{p}}{E{T}_{p}}+{{\rm{\varepsilon }}}_{n}\frac{dn}{n}$$

where ε_*P*_, ${{\rm{\varepsilon }}}_{E{T}_{p}}$, ε_*n*_ represent the *P*, *ET*_*p*_ and catchment landscape elasticity of *Q*, given by ref. 47:

13 $${{\rm{\varepsilon }}}_{P}=\frac{dQ/Q}{dP/P}\,{{\rm{\varepsilon }}}_{E{T}_{p}}=\frac{dQ/Q}{dE{T}_{p}/E{T}_{p}}\,{{\rm{\varepsilon }}}_{n}=\frac{dQ/Q}{dn/n}$$

Suppose that a, b, c are the value of ε_*P*_, ${{\rm{\varepsilon }}}_{E{T}_{p}}$, ε_*n*_, respectively. ε_*P*_(${{\rm{\varepsilon }}}_{E{T}_{p}}$) represents that a 1% increase in *P*(or *ET*_*p*_) would increase *Q* by ${\rm{a}}$% (or decrease *Q* by ${\rm{b}}$%). Similarly, ε_*n*_ represents that a 1% increase in *n* would decrease *Q* by ${\rm{c}}$%. Three Budyko equations are used in this study and elasticity coefficients of *P*, *ET*_*p*_ and catchment landscape parameter are given in Table 6.

**Table 6:** Estimations of annual actual evapotranspiration and elasticity coefficient based on the Budyko hypothesis.

Source(Year)	Equation	Elasticity coefficient
Budyko (1974)	$\frac{E{T}_{a}}{P}=\sqrt{\frac{E{T}_{p}}{P}\,\tanh (\frac{P}{E{T}_{p}})[1-\exp (-\frac{E{T}_{p}}{P})]}$	${{\rm{\varepsilon }}}_{P}=1+\frac{0.5\phi {[\phi \tanh (\frac{1}{\phi })(1-{e}^{-\phi })]}^{-0.5}[(\tanh (\frac{1}{\phi })-\frac{1}{\phi }{{\rm{sech}}}^{2}(\frac{1}{\phi }))(1-{e}^{-\phi })+\phi \,\tanh (\frac{1}{\phi }){e}^{-\phi }]}{1-{[\phi \tanh (\frac{1}{\phi })(1-{e}^{-\phi })]}^{0.5}}$
		$${{\rm{\varepsilon }}}_{E{T}_{p}}=\frac{0.5\phi {[\phi \tanh (\frac{1}{\phi })(1-{e}^{-\phi })]}^{-0.5}[(\tanh (\frac{1}{\phi })-\frac{1}{\phi }{{\rm{sech}}}^{2}(\frac{1}{\phi }))(1-{e}^{-\phi })+\phi \,\tanh (\frac{1}{\phi }){e}^{-\phi }]}{{[\phi \tanh (\frac{1}{\phi })(1-{e}^{-\phi })]}^{0.5}-1}$$
Fu *et al*. (1981)	$\frac{E{T}_{a}}{P}=1+\frac{E{T}_{p}}{P}-{[1+{(\frac{E{T}_{p}}{P})}^{m}]}^{\frac{1}{m}}$	${{\rm{\varepsilon }}}_{P}=\frac{{(1+{\phi }^{m})}^{(\frac{1}{m}-1)}}{{(1+{\phi }^{m})}^{(\frac{1}{m})}-\phi }{{\rm{\varepsilon }}}_{E{T}_{p}}=\frac{{\phi }^{m}(1+{\phi }^{m})(\frac{1}{m}-1)-\phi }{{(1+{\phi }^{m})}^{(\frac{1}{m})}-\phi }$
		$${{\rm{\varepsilon }}}_{m}=-{(1+{\phi }^{m})}^{(\frac{1}{m})}(\frac{\mathrm{ln}(1+{\phi }^{m})}{{m}^{2}}-\frac{{\phi }^{m}\,\mathrm{ln}(\phi )}{m(1+{\phi }^{m})})$$
Choudhury (1999)	$\frac{E{T}_{a}}{P}=\frac{E{T}_{p}}{{({P}^{n}+E{{T}_{p}}^{n})}^{\frac{1}{n}}}$	${{\rm{\varepsilon }}}_{P}=\frac{{(1+{\phi }^{{n}_{0}})}^{(1/{n}_{0}+1)}-{\phi }^{{n}_{0}+1}}{(1+{\phi }^{{n}_{0}})[{(1+{\phi }^{{n}_{0}})}^{(1/{n}_{0})}-\phi ]}{{\rm{\varepsilon }}}_{E{T}_{p}}=\frac{1}{(1+{\phi }^{{n}_{0}})[1-{(1+{\phi }^{-{n}_{0}})}^{(1/{n}_{0})}]}$
		$${{\rm{\varepsilon }}}_{{n}_{0}}=\frac{\mathrm{ln}(1+{\phi }^{{n}_{0}})+{\phi }^{{n}_{0}}\,\mathrm{ln}(1+{\phi }^{-{n}_{0}})}{{n}_{0}(1+{\phi }^{{n}_{0}})[1-{(1+{\phi }^{-{n}_{0}})}^{(1/{n}_{0})}]}$$

## Electronic supplementary material

Supplementary Information
