# Supplementary material for: Identification of potential impacts of climate change and anthropogenic activities on streamflow alterations in the Tarim River Basin, China
Source: Sci Rep. 2017 Aug 15;7:8254. doi: 10.1038/s41598-017-09215-z (PMC5557968; doi:10.1038/s41598-017-09215-z)
Supplement: Supplementary file 1 — Supplementary Information [file 41598_2017_9215_MOESM1_ESM.pdf]

Identification of potential impacts of climate change and anthropogenic activities on streamflow alterations in the Tarim River Basin, China

Lianqing Xue, Fan Yang, Changbing Yang, Xinfang Chen, Luochen Zhang, Yixia Chi and Guang Yang

Supplementary Information (SI)

SI Figure legends

Figure S1. Plots of land use and vegetation cover in the Tarim River Basin in 1980, 1990, 2000, 2008, and 2015(Note: the data set is provided by Data Center for Resources and Environmental Sciences, Chinese Academy of Sciences (RESDC) (http://www.resdc.cn), and the map was generated using reclassify tool in ESRI's ArcGIS (version 10.1).)

Figure S2. Plots of water consumption at b) water consumption of three plain reservoirs (WCPR) and c) oasis water consumption (OWC), the ratio of water consumption of three plain reservoirs in total water consumption (RWCPR) at a) and the aridity index (ET_P/P) at d) (Note: the data set is provided by the Tarim River Basin Administration, the aridity index and oasis water consumption from 1960 to 2011 and water consumption of three plain reservoirs from 1980 to 2006.)

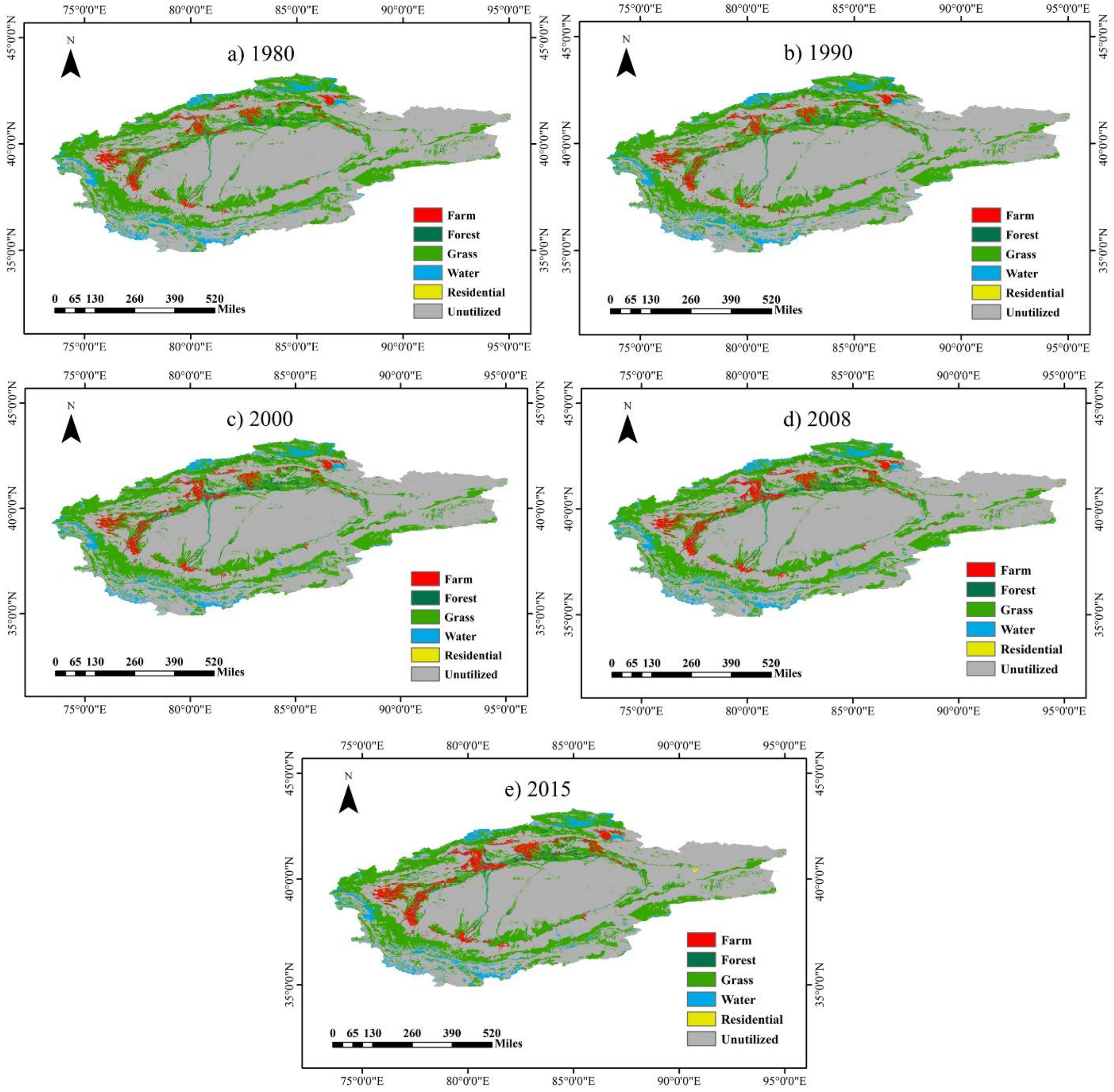
a) 1980
b) 1990
c) 2000
d) 2008
e) 2015
N
Farm
Forest
Grass
Water
Residential
Unutilized
0 65 130 260 390 520
Miles
75°0'0"E
80°0'0"E
85°0'0"E
90°0'0"E
95°0'0"E
45°0'0"N
40°0'0"N
35°0'0"N

Figure S1.

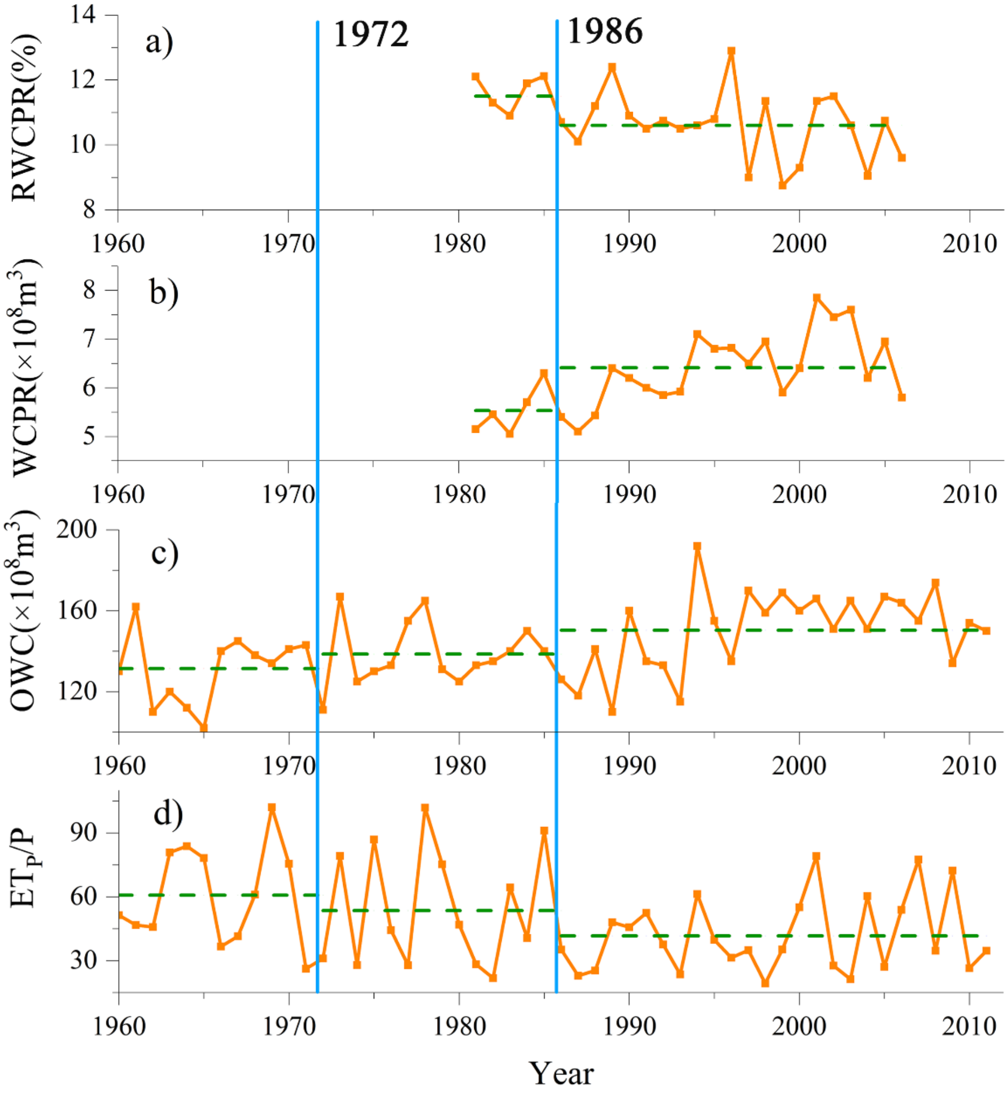
a)
1972
1986
RWCPR(%)
b)
WCPR($\times 10^8 m^3$)
c)
OWC($\times 10^8 m^3$)
d)
ET_P/P
Year

Figure S2.
